# Recent Advances in Catalytic Oxidation of Organic Sulfides: Applications of Metal–Ionic Liquid Catalytic Systems

**DOI:** 10.3389/fchem.2021.798603

**Published:** 2022-02-28

**Authors:** Xiao Bing Liu, Qi Rong, Jin Tan, Chen Chen, Yu Lin Hu

**Affiliations:** ^1^ College of Chemistry and Chemical Engineering, Jinggangshan University, Ji’an, China; ^2^ Key Laboratory of Inorganic Nonmetallic Crystalline and Energy Conversion Materials, College of Materials and Chemical Engineering, China Three Gorges University, Yichang, China; ^3^ College of Environmental and Chemical Engineering, Jiangsu University of Science and Technology, Zhenjiang, China

**Keywords:** ionic liquid, metal, oxidation of organic sulfides, oxidative desulfurization, application, recent advance

## Abstract

Catalytic oxidation of organic sulfides is of considerable significance in industrial chemistry and fuel industry. Therefore, numerous methods have been developed for the oxidation. Metal-containing ionic liquid-based catalysts can catalyze the selective oxidation reactions and are highly used in chemical processes, which have also been used as effective solvents, reaction media, extractants, and catalysts for the oxidation of organic sulfides including oxidative desulfurization of fuel oil. Recently, much attention is being drawn to the preparation of heterogenous catalysts based on the immobilization of metal- or nonmetal-containing ILs on diverse solid supports, which can be easily separated after the completion reaction and recycled. Therefore, there is still an increasing interest in developing new and efficient catalytic procedures for the oxidation of organic sulfides. In this review, we have outlined the recent advances in catalytic oxidation of organic sulfides including oxidative desulfurization of fuel oil. The versatilities and adaptabilities of metal–ionic liquid catalytic systems in the selective oxidation of sulfides are considered a powerful research field in these transformations.

## Introduction

Ionic liquids (ILs) have been considered an encouraging class of functional and green materials because of their unique properties such as non-volatility, non-flammability, adjustable structure, thermal and chemical stability, and nonexplosion. As a result, ILs are widely used in catalytic reaction and separation and can be used as reaction solvents, reaction media, and catalysts in catalytic reactions (Rajeswari et al*.*, 2016; Kore et al., 2017; Chiarotto et al., 2018; Lethesh et al., 2019; Raiguel et al., 2020; Nasrollahzadeh et al., 2021). In many cases, for easy separation and recyclable utilization, functional ILs have attracted considerable attention in many fields such as immobilized ILs catalysts, which could transfer the traditional catalytic reactions from homogenous catalysis to a type of heterogenous catalysis, and no additional metal-containing catalysts are needed. Recently, the immobilization of ILs onto various polymeric and inorganic solid supports for the formation of functional-supported ILs has attracted a surge of interest in industrially important catalytic processes. Such supported IL heterogenous catalytic systems not only reduce cost and enhance catalytic efficiency (high catalytic activity owing to a uniform distribution of ionic liquid-active species) but also facilitate catalyst separation and reutilization (they can be easily separated from the reaction products for further reusability). Various novel concepts of efficient and recyclable heterogenous catalytic systems based on supported ILs have been developed, which have been designed via the immobilization of metal- or nonmetal-containing ILs on diverse solid supports, which served as highly efficient catalysts in catalytic processes combining the unique properties of ILs and the attractive features of easy separation and recyclability (Xin and Hao, 2014; Marinkovic et al., 2019; Gupta et al., 2020).

Catalytic oxidation of organic sulfides is of considerable significance in industrial chemistry and fuel industry. The oxidations of sulfides to sulfoxides or sulfones are powerful reactions in industrial chemistry since the products sulfoxides or sulfones are important intermediates for the synthesis of valuable intermediates, pharmaceuticals, and natural products. Recently, the oxidation reactions with environmental-friendly oxidants using various metal-based catalysts have been reported for this transformation (Gonçalves et al., 2018; Zhang and Qi, 2018; Zhao et al., 2020). Deep desulfurization of fuel oil has attracted wide interest because of more stringent legislation and an increasing need for environmental protection. Therefore, numerous methods have been developed for the deep desulfurization of fuel oil, and a powerful method constitutes the oxidative desulfurization (ODS). In a typical ODS process, organic sulfides of fuels, such as DBT and its derivatives, can be oxidized to their corresponding sulfoxides and sulfones, followed by extraction or adsorption to achieve deep desulfurization of fuels, and a number of catalytic systems have been reported for the technology (Zhao and Baker, 2015; Bhutto et al., 2016; Yang et al., 2017; Ren et al., 2019; Wang et al., 2019; Mohumed et al., 2020). However, because of the drawbacks associated with the use of large amount of catalyst, difficulties in the separation, and catalyst recycling, new and efficient catalysts are being demanded.

In order to solve the problems described previously, the concept of metal–ionic liquid catalytic systems is being established, which could catalyze many reactions and are highly used in catalytic selective oxidation processes (Ternois et al., 2007; Venkat Reddy and Verkade, 2007; Baciocchi, et al., 2010; Zhang et al., 2012; Zhang et al., 2013; Carrasco et al., 2014; Rostamnia et al., 2016; Dai et al., 2017; Bayat et al., 2018). In recent research studies, numerous articles about ODS with ILs have been reported. Some functionalized ILs can act as both catalysts and extractive reagents, which combined the extraction with oxidative desulfurization (ECODS) (Juliao, et al., 2016; Wang, et al., 2017a; Jiang, et al., 2019a). For this reason, we emphasize the removal of sulfides being achieved by the oxidation process. Very recently, many studies have progressed on preparation and application of metal-containing ionic liquid-based catalysts including supported ionic liquids. Based on the aforementioned summarizations and inspired by the reports on the development of metal-containing ionic liquid-based catalysts in selective oxidation, which has attracted extensive attentions, it is a hot spot to study how to apply the prepared metal-containing ionic liquid-based catalysts in the catalytic oxidation of organic sulfides and oxidative desulfurization. In this review, we explore the current trends in the research directions toward the catalytic oxidation of organic sulfides and oxidative desulfurization in the presence of metal-containing ionic liquid-based catalytic systems, in which ILs and supported ILs are used as solvents, extractants, reaction media, catalysts, or their combinations during catalytic processes. The purpose of this review is to summarize first the facile route of the metal–ionic liquid catalytic systems for the efficient oxidation of organic sulfides including oxidative desulfurization of fuel oil, which would provide a new idea for the selective oxidation of sulfides with a strong connection to the chemical industry.

## Oxidation of Sulfides to Sulfones or Sulfoxides

**SCHEME 1:**
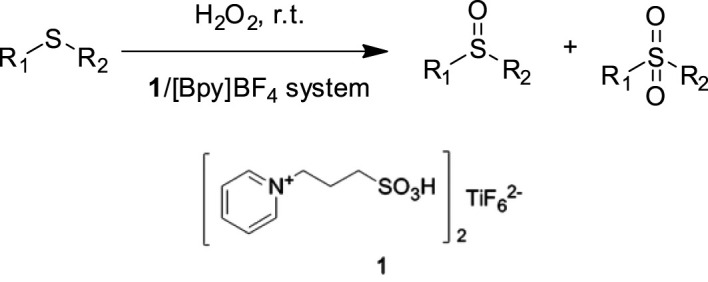
Sulfoxidation of sulfides with H_2_O_2_ in the presence of 1/(Bpy)BF_4_ catalytic system (Wang et al., 2012).

Wang et al*.* prepared a bifunctional ionic liquid bis-[N-(propyl-1-sulfoacid)-pyridinium] hexafluorotitanate **1** and studied its catalytic performances in the selective sulfoxidation of sulfides with H_2_O_2_ in (Bpy)BF_4_ (Wang et al., 2012). It was found that the catalytic system could efficiently catalyze the sulfoxidation, and a series of sulfides with different electronic and steric effects could be efficiently converted into the corresponding sulfoxides with 83–97% selectivity and 79–98% conversion at room temperature. Furthermore, for practical application, the request for easily separable and recyclable catalysts is driven by economic considerations and environmental concerns. Upon completion of the reaction, the IL catalytic system can be easily recovered and reused for six cycles without significant loss of its activity (Scheme 1).

**SCHEME 2:**
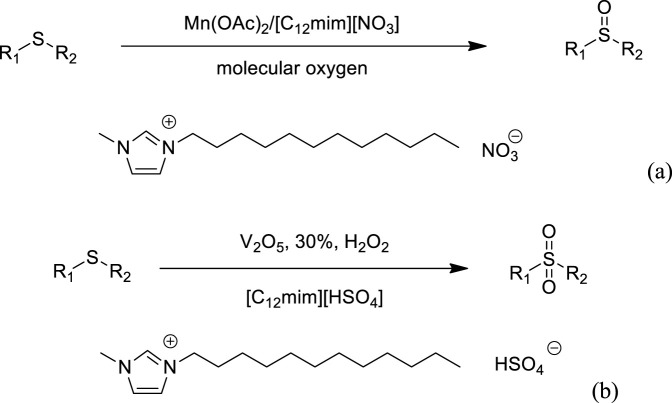
**(A)** Catalytic oxidation of sulfides to sulfoxides (Hu et al., 2014a). **(B)** Catalytic oxidation of sulfides to sulfones (Hu et al., 2014b).

Hu et al. developed an efficient and green method for the aerobic oxidation of sulfides to sulfoxides catalyzed by manganese acetate [Mn(OAc)_2_] in the ionic liquid reaction media [(C_12_mim)(NO_3_)] system (Scheme 2A). The reactions afford the target products in good to high yields, and no over-oxidation was observed (Hu et al., 2014a). In addition, the catalytic system could be typically recovered and reused for subsequent recycles with no appreciable decrease in yields and reaction rates. Hu et al. also introduced the oxidation of sulfides to sulfones with H_2_O_2_ catalyzed by vanadium pentoxide (V_2_O_5_) in the ionic liquid reaction media [(C_12_mim)(HSO_4_)] system (Scheme 2B). The products can be easily separated, and the catalytic system can be typically recovered and reused without loss of catalytic activity (Hu et al., 2014b).

**SCHEME 3:**
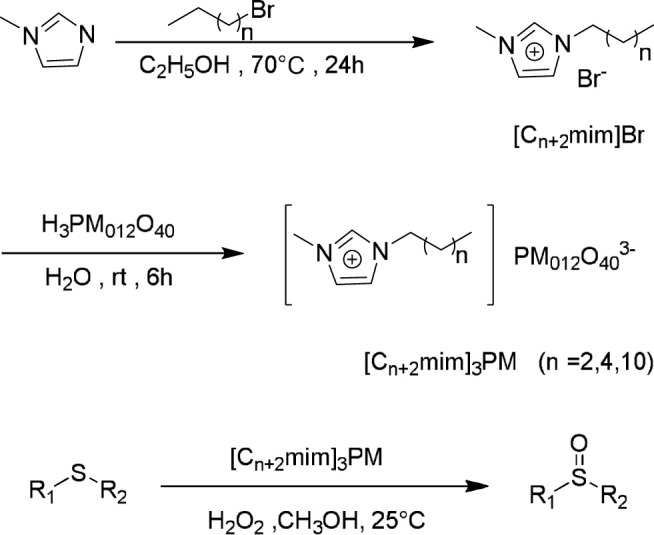
Synthesis of the imidazolium IL POM salts (C_n+2_mim)_3_PM and their catalytic oxidations of sulfides with H_2_O_2_ (Zhao, et al., 2012).

Zhao et al. reported the synthesis of a type of ionic liquid-based polyoxometalate (POM) salts through anion-exchange of imidazolium IL precursors with various Keggin POMs. The resultant imidazolium POM salts were shown to be highly effective catalysts for the selective oxidation of a variety of sulfides with aqueous H_2_O_2_ (Zhao, et al., 2012). The notable advantages of this method are high catalytic activity, convenient recovery, steady reuse, simple work-up, flexible composition, and mild reaction conditions. The preparation of the catalyst/reaction media dual role of the imidazolium ionic liquid-based POM salts and the catalytic oxidation procedure are shown in Scheme 3.

Rafiee et al. synthesized a type of Keggin-structured polyoxometalate-based ionic liquids by a two-step reaction including sulfonate-functionalized cations with Keggin-structured POM. The resultant ionic liquids, POM-ILs, were employed as a catalyst for the selective oxidation of sulfides to the corresponding sulfoxides (Rafiee and Mirnezami, 2014). The results showed that the catalyst PhPyBs-PW exhibited excellent activity in the oxidation reaction with good to high yields (70–98%) for the sulfide products. In addition, the catalyst could be easily recovered and reused for five runs with no significant loss of catalytic activity (Supplementary Scheme S1).

Bigi et al. developed a procedure for the enantioselective oxidation of methyl phenyl sulfide with CH_2_Cl_2_ as a solvent and UHP as an oxidant using trihexyltetradecylphosphonium ionic liquids {[P_6 6 6 14_]_2_[WO_2_(*S*-binol)_2_], [P_6 6 6 14_]_2_[WO_2_(*S*-mand)_2_]} containing tungsten(VI)-2,2′-(*S*)-binaphthol complex anions as efficient and recyclable catalysts under mild reaction conditions (Bigi, et al., 2011). The results showed that the catalytic performance of 5% sulfoxide yield, 100% selectivity, and 95% enantiomeric excess was obtained for the (P_6 6 6 14_)_2_[WO_2_(*S*-binol)_2_] catalyst, while 10% sulfoxide yield, 100% selectivity, and 95% enantiomeric excess were observed in the presence of (P_6 6 6 14_)_2_[WO_2_(*S*-mand)_2_]. The structures of tungsten(VI) metal center bearing chiral ligand anions and the catalytic oxidation of methyl phenyl sulfide are shown in Supplementary Scheme S2.

Fareghi-Alamdari et al. reported the synthesis of a novel Keplerate anions-based ionic liquid catalyst (NH_4_)_2_(MimAm)_40_[Mo_132_O_372_(CH_3_COO)_30_(H_2_O)_72_] (Mo132-MimAM) through the self-assembly strategy. The resultant Keplerate anions-based ionic liquid was shown to be a green, highly efficient, and reusable catalyst for the selective oxidation of a variety of sulfides with H_2_O_2_ to sulfoxides (Fareghi-Alamdari, et al., 2017). The notable advantages of this method are high catalytic activity, good-to-high conversions (88–100%), excellent chemoselectivity (94.5–99%), simple work-up, and mild reaction conditions. In addition, the catalyst of ionic liquid compounds based on Keplerate polyoxometalates could be easily separated from the reaction mixture by facile filtration and could be well retained in seven recycling oxidations, with a negligible deactivation in catalytic activity during each cycle (Supplementary Scheme S3).

**SCHEME 4:**
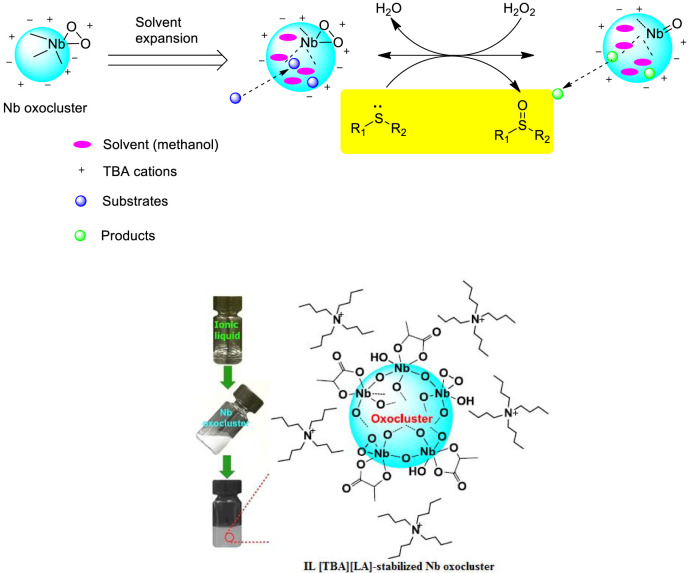
Reaction route for selective oxidation of sulfides with the Nb oxocluster catalyst (Zhou et al., 2019).

Zhou et al*.* prepared a new class of IL-stabilized Nb oxoclusters by coordination stabilization with carboxylate ionic liquids and studied their catalytic performances in the selective oxidation of sulfides with H_2_O_2_ in CH_3_OH solvent (Zhou et al., 2019). It was found that the ionic liquid tetrabutylammonium lactate [(TBA)(LA)]-stabilized Nb oxoclusters [Nb-OC@(TBA)(LA)] were uniformly dispersed with an average particle size of 2–3 nm and could efficiently catalyze the selective oxidation, and a number of sulfides could be efficiently converted into the corresponding sulfoxides or sulfones by adjusting the equivalent of H_2_O_2_, reaction time, and reaction temperature. Interestingly, the results also confirmed that sulfoxides could be produced almost stoichiometrically, and sulfones could be produced with excellent selectivity under higher temperatures (50 or 60 °C) and more amounts of H_2_O_2_ oxidant (Scheme 4). Furthermore, it was found that Nb-OC@(TBA)(LA) could be reused for at least five consecutive cycles with high selectivity to sulfoxide although the activity had a slight decrease possibly due to the slight aggregation of the catalyst.

Rafiee et al. reported the selective oxidation of sulfides with H_2_O_2_ as an oxidant in the presence of organic–inorganic poly(4-vinylpyridine)-supported ionic liquid catalyst PVPyPSPMo_10_V_2_. A range of aryl sulfides were smoothly converted into the corresponding sulfoxides with high yields (95–98%) in short reaction times of 2–12 min (Rafiee and Shahebrahimi, 2017). The organic–inorganic hybrid polyionic liquid-based polyoxometalate was achieved by a three-step reaction. The first step consisted of the synthesis of 4-vinylpyridine propane sulfone via the reaction of 4-vinyl pyridine with 1,3-propanesultone, followed by the polymerization for the synthesis of poly 4-vinylpyridine propane sulfone, and in the third step PVPyPSPMo_10_V_2_ was synthesized by combining these polymers with H_5_PMo_10_V_2_O_40_ (Supplementary Scheme S4). Reusability of the catalyst PVPyPSPMo_10_V_2_ is an important advantage when it is introduced for practical application in catalytic processes. It was found that the catalyst could be reusable at least four times, and the yield of 98% for the first run to 90% for the fourth run was observed.

Doherty et al. developed a procedure for the one-pot synthesis of sulfoxides via the selective oxidation of sulfides with ethanol solvent and mobile phase using styrene-based peroxotungstate-modified polymer-immobilized imidazolium ionic liquids {PO_4_[WO(O_2_)_2_]_4_}@ImPIILP as powerful and recyclable catalysts under mild reaction conditions (Doherty, et al., 2016). A series of aryl sulfides was subjected for the sulfoxide production under the optimized reaction conditions with good conversions and high selectivities. The results also showed the excellent stability and reusability nature of the heterogenous catalyst {PO_4_[WO(O_2_)_2_]_4_}@ImPIILP, which could be retained for 12 recycling oxidations (Supplementary Schemes S5, S6).

**SCHEME 5:**
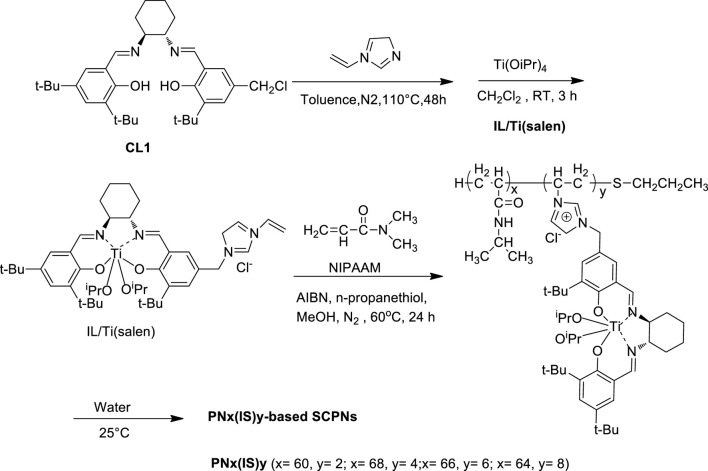
Schematic representation of synthesis and self-folding of PN_x_(IS)_y_ (Zhang Y et al., 2017).

Zhang et al. synthesized a series of enzyme-inspired SCPNs-containing vinylimidazolium ionic liquid-modified chiral salen Ti^IV^ complex (Scheme 5), and these resultant SCPNs were tested as efficient catalysts in the selective asymmetric sulfoxidation of a variety of sulfides in the water solvent with H_2_O_2_ oxidant under mild conditions. The results showed that PN_68_(IS)_4_ demonstrated the most excellent catalytic performance, and a range of aryl sulfides were smoothly converted into the corresponding sulfoxides with high conversions (>99%) and selectivities (95–99%). In addition, the catalysts could be easily separated from the aqueous system and be steadily reused in recycling oxidations due to the thermo-responsive property (Zhang L. et al., 2017).

Pourjavadi et al. prepared a new heterogenous catalytic system by immobilization of tungstate ions on cross-linked poly(ionic liquid) nanogel and studied its catalytic performance in the selective oxidation of sulfides to sulfoxides with H_2_O_2_ under solvent-free reaction conditions (Pourjavadi et al., 2015). A number of sulfides could be efficiently converted into the corresponding sulfoxides in high yields (83–98%) and conversions (85–99%). Moreover, the heterogenous catalyst of poly(ionic liquid) nanogel with tungstate anions could be easily recovered and reused for at least eight cycles without any loss of catalytic activity (Supplementary Scheme S7).

Doherty et al. prepared novel heterogenous polymer-immobilized ionic liquid {PO_4_[WO(O_2_)_2_]_4_}@PIILP *via* anion exchange, which was employed as an efficient catalyst for the selective oxidation of sulfides under mild conditions (Doherty et al., 2015). It was found that the catalyst could efficiently catalyze the oxidation with good conversions (88–99%) and high selectivities for either sulfoxide (88–98%) or sulfone (86–100%). In addition, the peroxometalate-based polymer-immobilized ionic liquid phase catalyst could be easily used for the consecutive reaction process for 8 h under continuous flow operation without any loss of catalytic activity (Supplementary Scheme S8).

Tarkhanova et al. synthesized a family of silica-supported Cu- and Mo-containing imidazolium ionic liquids, which were tested as an active and green catalyst for selective oxidation of diethyl sulfide and methyl phenyl sulfide (Tarkhanova, et al., 2016). The results showed that the silica-supported Cu-containing imidazolium ionic liquid exhibited the best catalytic performance for the diethyl sulfide oxidation with atmospheric oxygen, while the silica-supported Mo-containing imidazolium ionic liquid was the most active and stable catalyst for the methyl phenyl sulfide oxidation with hydrogen peroxide (Supplementary Scheme S9). In addition, the catalyst could be easily reused for at least three successive cycles.

Tarkhanova et al. also reported the synthesis of mineral-immobilized ionic liquids based on molybdenum- and tungsten-containing heteropolyacids, which were tested as an efficient heterogenous catalyst for selective oxidation of thiophene with hydrogen peroxide oxidant in an isooctane solvent (Tarkhanova et al., 2017) (Supplementary Scheme S10). The results showed that the alumina-supported PMA-based PMo/Al_2_O_3_ catalyst exhibited the best catalytic performance for the oxidation and could be easily reused for successive cycles (Supplementary Figure S1).

**SCHEME 6:**
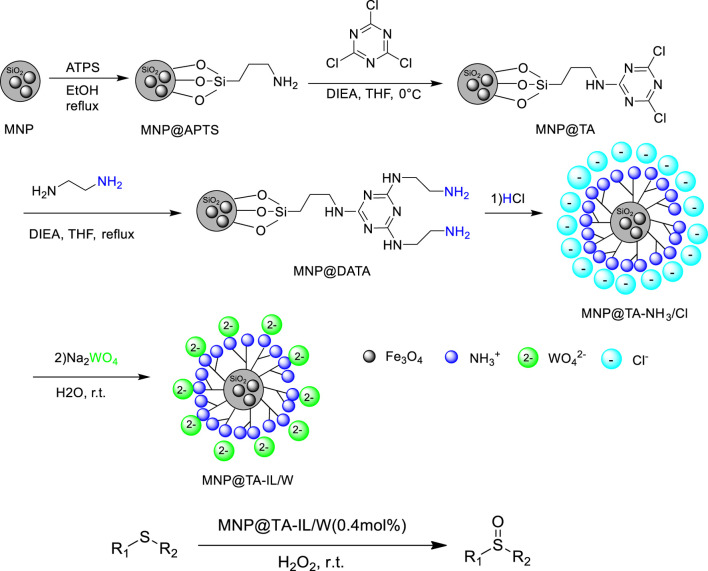
Preparation of MNP@TA-IL/W and catalytic oxidation of sulfides to sulfoxides (Hosseini et al., 2018).

Hosseini et al. reported the selective oxidation of sulfides with H_2_O_2_ as an oxidant catalyzed by magnetic-supported triazine-based ionic liquid MNP@TA-IL/W at room temperature. Various sulfides could be smoothly converted into the corresponding sulfoxides with high conversions (83–99%) and excellent yields (95–99%) in 1–3 h (Hosseini et al., 2018). The magnetic-supported triazine-based ionic liquid was prepared by a five-step reaction. The first step consisted of the synthesis of amine-functionalized magnetic nanoparticles (MNP@APTS) via the reaction of APTS with silica-coated Fe_3_O_4_ nanoparticles, the second step included the functionalization of MNP@APTS with TCTA for the synthesis of MNP@TA, and in the third step MNP@DATA was synthesized by the reaction of MNP@TA with DIEA, and then HCl was added for the synthesis of MNP@TA-NH_3_/Cl, followed by chloride exchange process with tungstate to give the catalyst (Scheme 6). In addition, the catalyst was magnetically recovered and reused for up to six runs without obvious decrease of activity and selectivity. More important, easy gram-scale oxidation of methyl phenyl sulfide and fast separation of the catalyst/product make the method economical and industrially applicable.

Li et al. reported novel modular polyoxometalate-layered double hydroxides Mg_3_Al-ILs-La(PW_11_)_2_ and used the catalyst for the selective oxidation of sulfides to sulfoxides with H_2_O_2_ as a green oxidant in methanol (Li T. et al., 2018). The designed catalyst demonstrated a high efficiency for the sulfoxidation, with the conversions and selectivities above 99 and 98%, respectively. The catalyst Mg_3_Al-ILs-La(PW_11_)_2_ could be easily recovered and reused at least five times without any loss of catalytic activity (Supplementary Scheme S11).

Karimi et al. synthesized a novel periodic mesoporous organosilica-supported ionic liquid (WO_4_^2−^@PMO-IL) and then employed it as a catalyst for the selective oxidation of sulfides to the corresponding sulfoxides (Karimi et al., 2015). They found that the tungstate supported on periodic mesoporous organosilica with the imidazolium framework catalyst could efficiently catalyze the oxidation with moderate to excellent yields (52–98%) and good to excellent selectivities (83–98%) (Supplementary Scheme S12).

**SCHEME 7:**
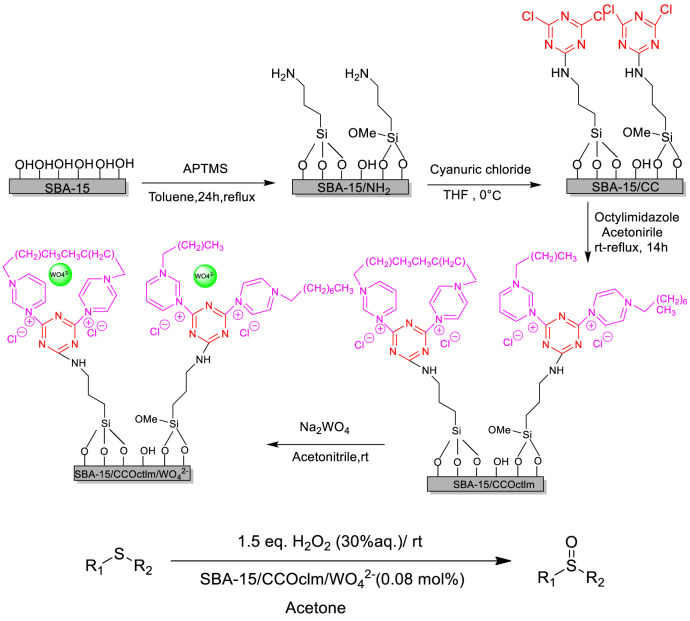
Pathways of WO _4_^2−^@SBA-15/IL fabrication and catalytic oxidation of organic sulfides (Sedrpoushan et al., 2018).

Sedrpoushan et al. prepared a new heterogenous catalyst (SBA-15/Im/WO_4_^2−^) and studied its catalytic performance in the selective oxidation of organic sulfides with H_2_O_2_ under neutral conditions. Various sulfides could be efficiently converted into the corresponding sulfoxides with good to excellent yields and selectivities (Sedrpoushan et al., 2018). Moreover, the supported ionic liquid catalyst could be easily recovered and retained its good activities for five recycles without any loss of catalytic activity. The tungstate-supported mesoporous silica SBA-15 with the imidazolium framework catalyst was prepared by a four-step reaction including the preparation of SBA-15/IL and chloride anion exchange with tungstate (Scheme 7).

Carrasco et al. prepared a mesoporous SBA-15-supported ionic liquid SBA-15 + ImCl + MoO_5_ by the reaction of 1-methyl-3-[3-(triethoxysilyl)propyl]-1H-imidazol-3-ium chloride with SBA-15 support and then followed by the immobilization of the oxodiperoxo–molybdenum complex (Carrasco et al., 2015). The supported ionic liquid was then investigated as a catalyst for the selective oxidation of sulfides to sulfoxides with 30% aqueous H_2_O_2_ oxidant at room temperature. Several sulfides including diphenylsulfide, methyl phenyl sulfide, methyl-p-tolylsulfide, 4-chlorothioanisole, 4-bromothioanisole, and ethylphenylsulfide could be efficiently converted into the corresponding sulfoxides in moderate to excellent conversions. Moreover, the heterogenous catalyst could be easily recovered and reused for ten cycles without any loss of catalytic activity (Supplementary Scheme S13).

Moaser et al. prepared a new heterogenous catalyst by immobilization of the molybdenum (VI)-based oxido–peroxido complex on periodic mesoporous organosilica-supported bipyridinium ionic liquid MoO(O_2_)_2_@Bipy-PMO-IL and studied its catalytic performance in the selective oxidation of sulfides to sulfoxides with H_2_O_2_ in water (Moaser et al., 2020). A number of aryl sulfides could be efficiently converted into the corresponding sulfoxides in good to high yields (75–91%) within a short reaction time of 30 min. Furthermore, the catalyst could be easily recovered and reused five times (Supplementary Scheme S14).

Hosseini-Eshbala et al. prepared a novel hybrid nanocatalyst (CMK-3-OctIm/MoO_4_^=^) by immobilization of molybdate ions (MoO42-) on octylimidazolium ionic liquid-modified ordered hexagonal mesoporous carbon CMK-3 (CMK-3/OctIm) and studied its catalytic performance in the selective oxidation of sulfides to sulfoxides with H_2_O_2_ in acetonitrile (Hosseini-Eshbala et al., 2020). Different sulfides with electron-donating and electron-withdrawing groups could be efficiently converted into the corresponding sulfoxides in good to high yields (80–96%) in short times of 7–9 min. Furthermore, the catalyst could be easily recovered and reused four times with no significant decrease in its activity (Supplementary Scheme S15).

Rajabi et al. developed an efficient method for the selective aerobic oxidation of sulfides to sulfoxides or sulfones catalyzed by tungstate-functionalized Brönsted acidic ionic liquid PMO-IL-WO_4_^2-^ with H_2_O solvent (Rajabi et al., 2021). It was found that the catalyst PMO-IL-WO_4_^2-^ can selectively produce sulfoxides or sulfones by running the reaction at room temperature or 50°C, respectively. A number of sulfides could be efficiently converted into the corresponding sulfoxides or sulfones with high yields (92–99%). Moreover, the catalyst could be easily recovered and reused eight times without loss of its activity (Supplementary Scheme S16).

## Deep Oxidative Desulfurization

Wang et al. prepared a series of pristine V_2_O_5_/SBA-15 composites and tested their catalytic performance for the oxidative desulfurization of fuels with molecular oxygen (O_2_) in ionic liquid (Bmim)BF_4_. The catalytic system showed a good catalytic activity in the oxidative desulfurization, and sulfur compounds in oils could be extracted into the IL phase and oxidized to their corresponding sulfones (Wang et al., 2017b). The results showed that the sulfur removal of dibenzothiophene could reach up to 99.3% (<3.5 ppm). In addition, the catalytic system can be retained for six cycles with no significant loss on sulfur removal (Supplementary Figure S2).

**SCHEME 8:**
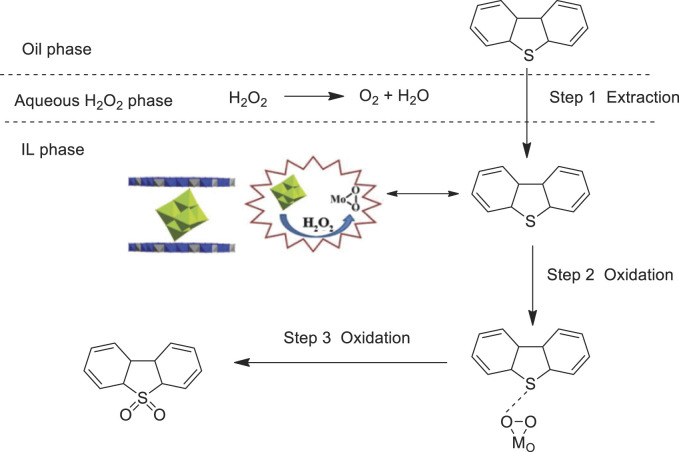
Catalytic mechanism of sulfur removal for DBT (Guo et al., 2018).

Guo et al. prepared a novel material Mg_3_Al-Mo_6_ by a one-pot hydrothermal method of (Mo_6_O_19_)^2−^ anion intercalation into layered double hydroxides and tested its catalytic performance for the oxidative desulfurization of fuels with H_2_O_2_ in ionic liquid (Bmim)PF_6_. The catalytic system showed superior catalytic activity in the oxidative desulfurization, and sulfur compounds in oils could be extracted into ionic liquid and converted to sulfones (Guo et al., 2018). The results showed that the sulfur removal of dibenzothiophene (DBT) and benzothiophene (BT) could reach up to almost 100% in 30 and 40 min, respectively. In addition, the polyoxomolybdate anion-intercalated layered double hydroxides catalyst can be easily recovered and reused for at least seven cycles with no obvious decrease in sulfur removal (Scheme 8).

Hao et al. prepared a novel polyoxometalates-based ionic liquid (PyPS)_3_(NH_4_)_3_Mo_7_O_24_ and employed it as a catalyst for the oxidation/extractive desulfurization of model and actual diesel with hydrogen peroxide oxidant in IL (Omim)BF_4_ extraction solvent (Hao et al., 2019). The results showed that the catalytic system (PyPS)_3_(NH_4_)_3_Mo_7_O_24_/(Omim)BF_4_ exhibited the unprecedented activity in the oxidative desulfurization with 99% sulfur-removal of DBT under quite mild conditions (Supplementary Scheme S17). Moreover, the catalytic system could be easily recovered and reused five times without significantly reducing the desulfurization activity.

**SCHEME 9:**
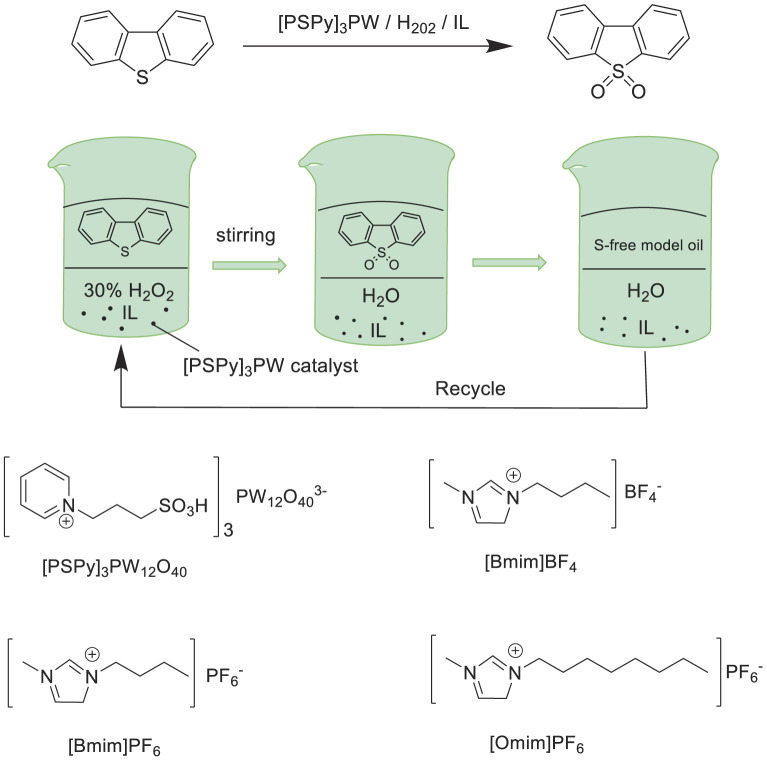
Structure of ILs and catalytic oxidation/extractive desulfurization of fuels.

Huang et al. prepared a new heteropolyanion-based ionic liquid (PSPy)_3_PW_12_O_40_ [(PSPy)_3_PW] and employed it as an effective catalyst for the oxidation/extractive desulfurization of fuels with hydrogen peroxide oxidant in ionic liquid (Omim)PF_6_ extraction solvent (Huang et al., 2010). The results showed that the catalytic system exhibited the excellent activity in the oxidative desulfurization with 99.4% sulfur-removal of DBT and 98.8% removal of 4,6-DMDBT under the optimal conditions in model oil (Scheme 9). Moreover, the catalytic system could be reused at least nine times without significant decrease in activity. Additionally, the sulfur removal of FCC fuel could be reduced from 360 ppm to 70 ppm with the catalytic system.

Saikia et al. reported a method for the oxidation of organic sulfur (aryls) components to sulfones with H_2_O_2_ in extractive coupled with catalytic oxidative desulfurization in the presence of HCOOH/H_2_O_2_ and V_2_O_5_ and ionic liquid 1-n-butyl-3-methylimidazolium tetrafluoroborate (IL_1_) or 1-n-butyl 3-methylimidazolium chloride (IL_2_) (Saikia et al., 2014). It was found that the removal of maximum total sulfur could reach 50.20%, the organic sulfur could reach 48.00%, and the ash could reach 70.37 wt% in this process. In addition, the ionic liquids could be recovered and subsequently reused for further desulfurization (Supplementary Scheme S18).

**SCHEME 10:**
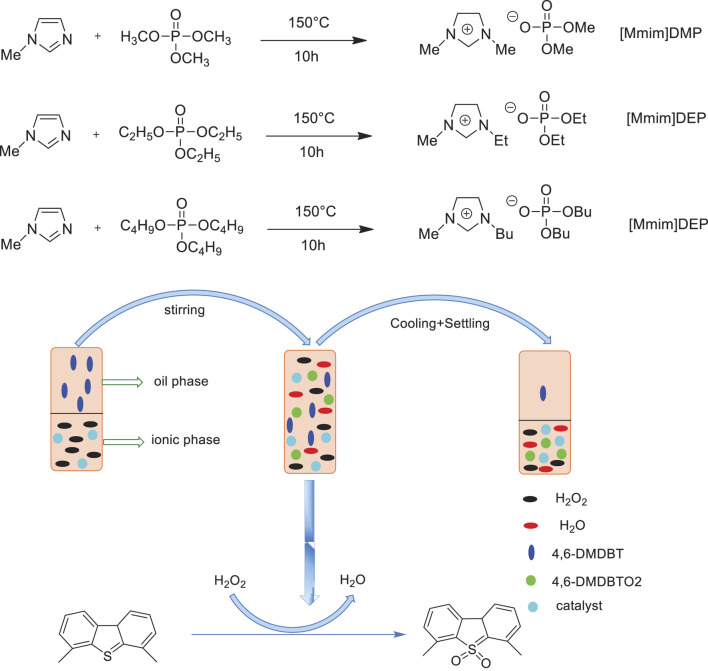
Synthesis of the ionic liquids and the catalytic process of extraction coupled with catalytic oxidation of 4,6-DMDBT.

Shao et al. prepared a series of imidazolium-based phosphoric ionic liquids [(Mmim)DMP, (Emim)DEP, (Bmim)DBP] and employed those in the extraction and catalytic oxidation desulfurization system (ECODS) from a model diesel fuel with the hexaammonium heptamolybdate tetrahydrate (NH_4_)_6_Mo_7_O_24_·4H_2_O) catalyst and 30%H_2_O_2_ oxidant (Shao et al., 2014). It was found that the catalytic system (NH_4_)_6_Mo_7_O_24_·4H_2_O/(Bmim)DBP exhibited the highest desulfurization activity with 89.2% S-removal of 4,6-DMDBT in model diesel fuel, which was markedly superior to mere solvent extraction with ionic liquid (9.29%) or catalytic oxidation without ionic liquid (5.34%) (Scheme 10). Moreover, the catalytic system could be reused at least six times with no significant decrease in activity.

Zhang et al. synthesized a kind of superbase-derived Lewis acidic ionic liquid with the protonated 1,5-diazabicyclo(4.3.0)-non-3-ene (DBN) cation and the ZnCl_2_-based complex anion (HDBN)Cl/nZnCl_2_ (Supplementary Scheme S19). (HDBN)Cl/nZnCl_2_ was shown to be effective catalysts for the extraction-combined oxidative desulfurization (ECODS) process of both model oil and real diesel with hydrogen peroxide (Zhang L. et al., 2017). The results showed that [HDBN]Cl/ZnCl_2_ exhibited the best activity in the oxidative desulfurization with complete removal of dibenzothiophene compound in oil. In addition, the catalyst (HDBN)Cl/ZnCl_2_ is recyclable and reusable and retains activity after five cycles with no noticeable changes in sulfur removal performance (Supplementary Figure S3).

**SCHEME 11:**
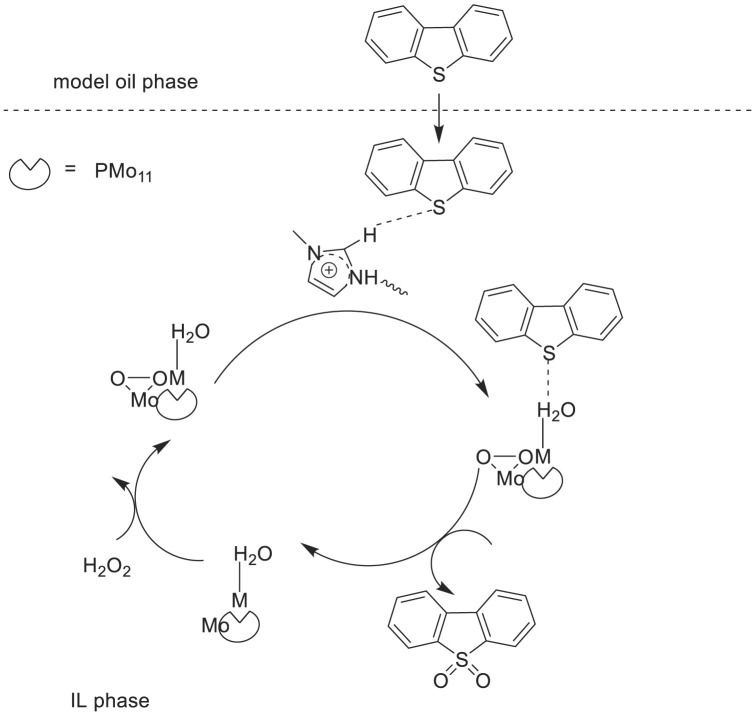
Catalytic mechanism of extractive and oxidation desulfurization (ECODS).

Li et al. synthesized a type of polyoxometalates-based ionic liquids (POM-ILs) (Bmim)_5_[PMo_11_M(H_2_O)O_39_] (M = Co^2+^, Ni^2+^, Zn^2+^, Mn^2+^) containing transition metal mono-substituted Keggin-type phosphomolybdates. Then, (Bmim)_5_[PMo_11_M(H_2_O)O_39_] was used as catalysts for the oxidation/extractive desulfurization of model oil with hydrogen peroxide oxidant in ILs extraction solvent (Li Y. et al., 2018). The results showed that the catalyst (Bmim)_5_[PMo_11_Co(H_2_O)O_39_] with [Omim]BF_4_ solvent exhibited the best catalytic activity in the oxidative desulfurization with 99.8%, 63%, and 85% sulfur-removal of DBT, BT, and 4,6-dimethyldibenzothiophene (4,6-DMDBT), respectively (Scheme 11). In addition, the catalytic system could be easily recovered and reused for at least eight cycles with good stability and high catalytic activity for consecutive desulfurization.

Wang et al. synthesized a type of Lewis acidic ionic liquid with alkylated 1,8-dia-zabicyclo[5.4.0]undec-7-ene (DBU) cation and the ZnCl_2_-based complex anion [ODBU]Cl/nZnCl_2_ (Supplementary Scheme S20). (ODBU)Cl/nZnCl_2_ was shown to be effective catalysts for the extraction-combined oxidative desulfurization (ECODS) process of both model oil and real diesel with 30%H_2_O_2_ oxidant (Wang et al., 2018). The results showed that (ODBU)Cl/3ZnCl_2_ exhibited the best catalytic activity in the oxidative desulfurization with 99.2% sulfur-removal (S-content reduced from 559.7 to 4.8 ppm) under mild conditions. In addition, the catalyst (ODBU)Cl/3ZnCl_2_ is recyclable and reusable and retains activity after six cycles with no noticeable changes in catalytic performance (Supplementary Figure S4).

**SCHEME 12:**
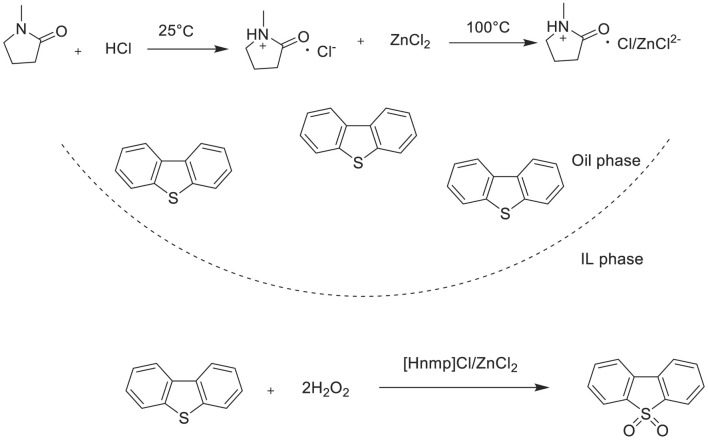
Synthesis of (Hnmp)Cl/ZnCl_2_ and catalytic oxidation desulfurization using Brønsted−Lewis acidic IL (Hnmp)Cl/ZnCl_2_ with H_2_O_2_ in this ODS system.

Chen et al. prepared a series of Brønsted–Lewis acidic ILs of N-methylpyrrolidonium zinc chloride[ (Hnmp)Clx/(ZnCl_2_)y, x:y from 2:1 to 1:2] and studied their catalytic performances for the oxidative desulfurization of both model diesel fuel and real FCC diesel fuel with 30%H_2_O_2_ oxidant (Scheme 12). In this catalytic system, these ILs could be used as both the extractant and catalyst; IL composition also has an important effect on sulfur removal efficiency. It was found that the ionic liquid (Hnmp)Cl/ZnCl_2_ showed the highest desulfurization activity with 99.9% S-removal in model diesel fuel (S-content can be reduced from 500 ppm to <1 ppm), and with 97.6% S-removal in FCC diesel fuel after five stages (S-content can be reduced to 5.3 ppm) (Chen et al., 2015). In addition, it was found that (Hnmp)Cl/ZnCl_2_ has a good recycling performance in the ODS process. After seven runs, the S-removal efficiency has a slight decline, which might be ascribed to the accumulation of the oxidation product (Supplementary Figure S5).

Xu et al. prepared a type of silica gel-supported ionic liquid (Bmim)CoCl_3_/SG *via* the simple sol–gel method and employed it as a heterogenous catalyst in the extraction and catalytic oxidation desulfurization system (ECODS) from a simulated fuel oil with oxone oxidant in ionic liquid (Bmim)BF_4_ (Xu et al., 2021). It was found that the extractive catalytic system (Bmim)CoCl_3_/SG/(Bmim)BF_4_ could achieve deep desulfurization with a desulfurization rate of 99.5% for DBT. Moreover, the desulfurization results revealed that the desulfurization of the three sulfur-containing substances followed the order of DBT *>* BT *>* 4,6-DMDBT (Supplementary Figure S6). Additionally, the catalytic system could be reused five times with no significant decrease in activity (all the desulfurization rate above 90%).

**SCHEME 13:**
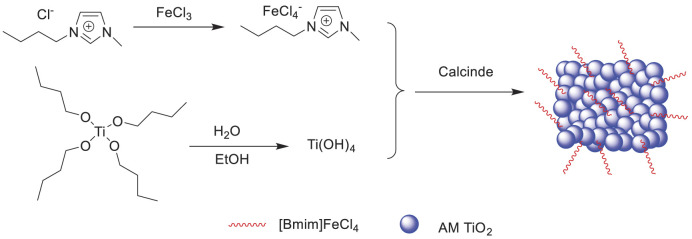
Preparation of the supported ionic liquid catalyst [Bmim]FeCl_4_/Am TiO_2_.

Xun et al. reported a method for the oxidation of sulfur compounds to sulfones with H_2_O_2_ in extractive coupled with catalytic oxidative desulfurization (ECODS) in the presence of supported ionic liquid [Bmim]FeCl_4_/Am TiO_2_ (Scheme 13. It was found that [Bmim]FeCl_4_ and Am TiO_2_ had a synergistic effect on the catalytic oxidation desulfurization (Xun et al., 2015). Furthermore, the catalyst could be easily recovered and reused 25 times with no significant decrease in its activity (Supplementary Figure S7).

**SCHEME 14:**
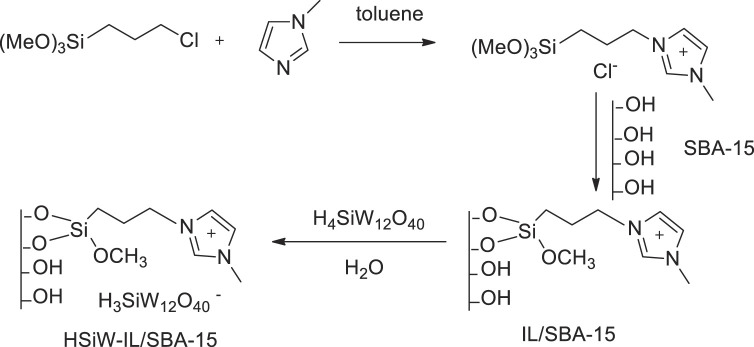
Synthetic process of HSiW-IL/SBA-15.

Yuan et al. prepared the SBA-15-supported silicotungstic acid ionic liquid HSiW-IL/SBA-15 *via* a covalent grafting method (Scheme 14). The supported catalyst showed excellent catalytic activity in the oxidative desulfurization of fuels with H_2_O_2_. The SBA-15-supported ionic liquid catalyst, 0.2HSiW-IL/SBA-15, was found to be more active than other HSiW different loading catalysts such as 0.05HSiW-IL/SBA-15, 0.1HSiW-IL/SBA-15, 0.2HSiW-IL/SBA-15, and 0.3HSiW-IL/SBA-15 (Yuan et al., 2016). The performance of the catalyst 0.2HSiW-IL/SBA-15 can be retained for eight experiments, and the sulfur removal still remained at 96.4% (Supplementary Figure S8).

Jiang et al. prepared two kinds of magnetic catalysts (IL/MMS-S and IL/MMS-L) by the immobilization of ionic liquid [(C_18_H_37_)_2_(CH_3_)_2_N]_3_PW_12_O_40_ on the small core-shell magnetic mesoporous silica (MMS-S) or large core-shell magnetic mesoporous silica (MMS-L) microspheres and tested their catalytic performance for the oxidative desulfurization of diesel fuel with H_2_O_2_ (Jiang et al., 2019b). Compared with the catalyst IL/MMS-L, the small size catalyst IL/MMS-S showed superior catalytic activity in the oxidative desulfurization, and the 4,6-DMDBT removal with the sulfur content decreasing to 9.2 ppm (Supplementary Figure S21). In addition, the catalytic system can be easily separated and reused at least eight times with no obvious decrease in sulfur removal activity. The recycling experiment of IL/MMS-S showed that the catalyst had outstanding stability.

Ding et al. prepared a novel SBA-15-supported iron-based redox ionic liquid (pmim)FeCl_4_-SBA-15 and employed it as a catalyst for the oxidation desulfurization of model oil with hydrogen peroxide oxidant in (Omim)BF_4_ extraction solvent (Ding et al., 2015). The results showed that the catalytic system (pmim)FeCl_4_-SBA-15/(Omim)BF_4_ exhibited the excellent activity in the oxidative desulfurization with 94.3% sulfur-removal of DBT under optimum conditions (Supplementary Scheme S22). Moreover, it was found that the different substrates of the ECODS selectivity followed the order of DBT > DT > BT in the catalytic system (Supplementary Figure S9). Additionally, (Omim)BF_4_ served as not only the extractant and the reaction media but also the co-catalyst in the system.

Xun et al. prepared a new few-layered graphitic carbon nitride (g-C_3_N_4_) supported quaternary phosphonium ionic liquid [(C_6_H_13_)_3_PC_14_H_29_]_3_PMo_12_O_40_ (C_14_PPMo IL) and employed it as a heterogenous catalyst for the oxidative desulfurization of model oil with hydrogen peroxide under solvent-free conditions (Xun et al., 2020). The results showed that the supported catalyst 5% C_14_PPMo/g-C_3_N_4_ exhibited extraordinary catalytic activity in the oxidative desulfurization with 100% sulfur-removal of DBT and 94.8% removal of 4,6-DMDBT after a 180-min reaction under mild conditions (Supplementary Figure S10). Moreover, the catalyst could be easily separated and reused six times with no obvious decrease in activity with the oxidative removal efficiency of 93.8% for DBT and 90.2% for 4,6-DMDBT.

**FIGURE 1:**
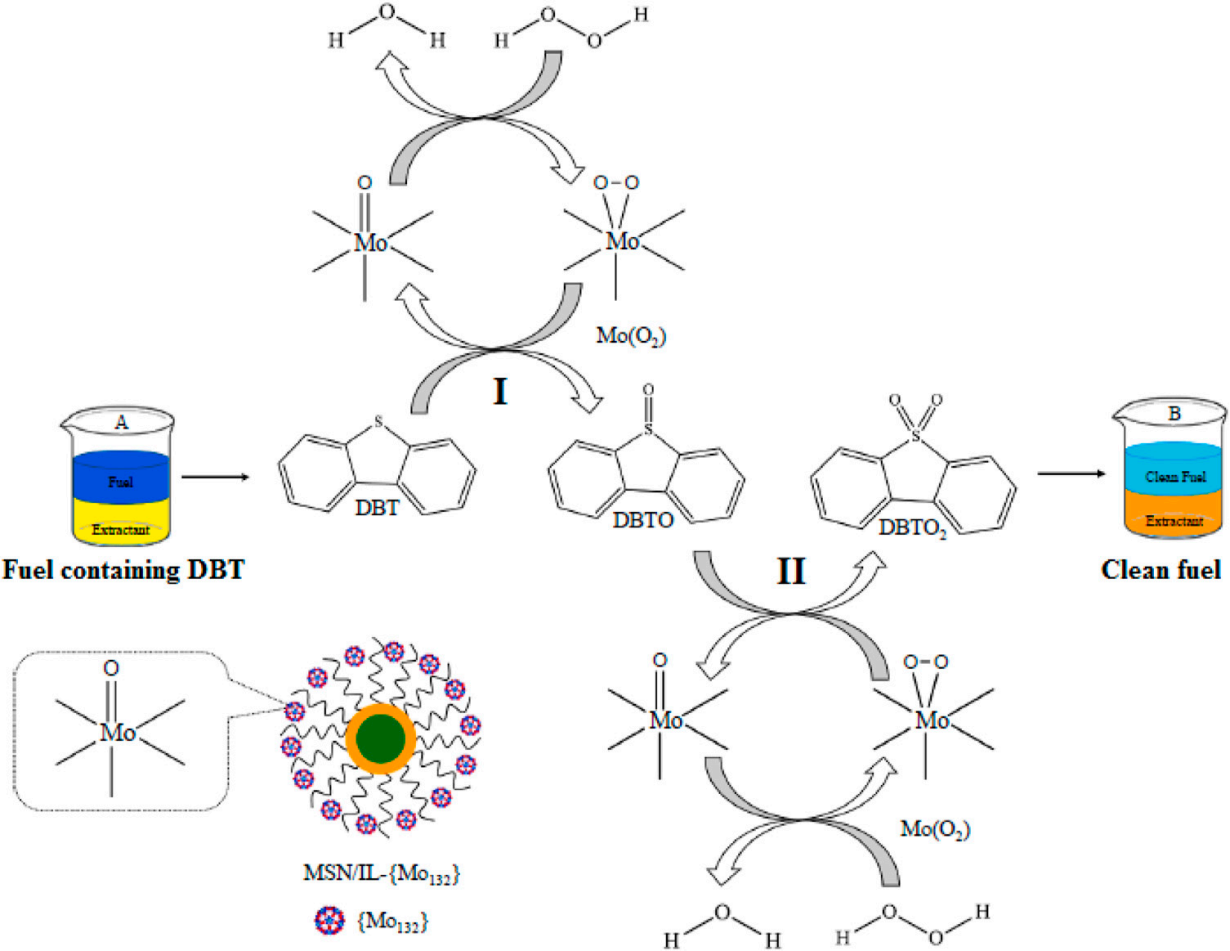
Catalytic oxidation of DBT using H_2_O_2_ over MSN/IL-(Mo132) catalyst **(A)** before and **(B)** after the ODS process (Mojaverian Kermani et al., 2021).

Kermani et al. prepared a novel magnetic silica-supported ionic liquid [MSN/IL-(Mo132)] *via* the immobilization of Keplerate nanoball iso-polyoxomolybdate (Mo_132_) on ionic liquid-functionalized magnetic silica nanoparticles (MSN/IL) and employed it as an effective catalyst for the deep oxidative desulfurization of a model fuel containing DBT with hydrogen peroxide as an oxidant (Mojaverian Kermani et al., 2021). The results showed that the catalytic system exhibited the excellent activity in the oxidative desulfurization with 99.97% sulfur-removal of DBT under optimal conditions (Figure 1). Moreover, the catalyst could be simply separated by an external magnetic field and reused four times without significant decrease in activity. This procedure has advantages such as simple procedure, high efficiency, easy recoverability, and recyclability, which was a green and efficient route toward the deep oxidative desulfurization.

## Conclusion and Outlooks

In summary, in this review, we have reported a serial of efficient procedures for the oxidation of organic sulfides including oxidative desulfurization of fuel oil. Various metal–ionic liquid-based catalytic systems having highly promising future prospects in the field of oxidation of organic sulfides have been investigated. It is to be expected that in future the scope and diversity of oxidation applications using ionic liquid-based catalysts will be further increasing; the possibilities of heterogenous-supported ionic liquid catalysts to reduce environmental pollution and to make experimental procedures simple and easy will be expanded. The traditional catalysts that have been used in oxidation reactions will be progressively replaced by green and environmental-friendly catalysts such as ionic liquids. However, the limitations of ILs such as the unknown toxicity and stability, recyclability, and economic problems blocked the industrial application in the selective oxidation. Future efforts in the development of novel and highly efficient ionic liquid-based catalysts for the selective oxidation of organic sulfides including oxidative desulfurization of fuel oil are still needed. The integration of ILs as an efficient solvent and catalyst with molecular oxygen as oxidant technologies, including functional IL synthesis, molecular oxygen activation, oxidation process, and catalyst recovery utilization is necessary to improve the actual efficiency. Furthermore, more efforts should be carried out on the study of oxidation mechanism, dynamic and thermodynamic models, reactor hydrodynamics, continuous-flow stirred tank oxidation reactor, and novel-supported IL catalysts for the solvent- and co-catalyst-free continuous-flow selective oxidation under mild conditions. Efforts in developing novel catalytic systems would probably offer new ways to find out which process can be ultimately applied in practical production. We believe that the development of efficient and economic ionic liquid-based catalytic systems will greatly broaden the future scopes and applications in the selective oxidation of organic sulfides.

## References

[B1] Baciocchi E. Chiappe C. Fasciani C. Lanzalunga O. Lapi A. (2010). Reaction of Singlet Oxygen with Thioanisole in Ionic Liquid−Acetonitrile Binary Mixtures. Org. Lett. 12, 5116–5119. 10.1021/ol102263w 21028790

[B2] Bayat Y. Shirini F. Goli-Jolodar O. (2018). Selective Oxidation of Sulfides to Sulfoxides Using Novel Nano Brönsted Dicationic Ionic Liquid as Effective Reagent under Grinding Conditions. J. Mol. Liquids. 265, 517–524. 10.1016/j.molliq.2018.06.036

[B3] Bhutto A. W. Abro R. Gao S. Abbas T. Chen X. Yu G. (2016). Oxidative Desulfurization of Fuel Oils Using Ionic Liquids: a Review. J. Taiwan Inst. Chem. Eng. 62, 84–97. 10.1016/j.jtice.2016.01.014

[B4] Bigi F. Nimal Gunaratne H. Q. Quarantelli C. Seddon K. R. (2011). Chiral Ionic Liquids for Catalytic Enantioselective Sulfide Oxidation. Comptes Rendus Chim. 14, 685–687. 10.1016/j.crci.2010.09.003

[B5] Carrasco C. J. Montilla F. Álvarez E. Mealli C. Manca G. Galindo A. (2014). Experimental and Theoretical Insights into the Oxodiperoxomolybdenum-Catalysed Sulphide Oxidation Using Hydrogen Peroxide in Ionic Liquids. Dalton Trans. 43, 13711–13730. 10.1039/c4dt01733a 25102034

[B6] Carrasco C. J. Montilla F. Bobadilla L. Ivanova S. Odriozola J. A. Galindo A. (2015). Oxodiperoxomolybdenum Complex Immobilized onto Ionic Liquid Modified SBA-15 as an Effective Catalysis for Sulfide Oxidation to Sulfoxides Using Hydrogen Peroxide. Catal. Today. 255, 102–108. 10.1016/j.cattod.2014.10.053

[B7] Chen X. Guo H. Abdeltawab A. A. Guan Y. Al-Deyab S. S. Yu G. (2015). Brønsted-Lewis Acidic Ionic Liquids and Application in Oxidative Desulfurization of Diesel Fuel. Energy Fuels. 29, 2998–3003. 10.1021/acs.energyfuels.5b00172

[B8] Chiarotto I. Mattiello L. Pandolfi F. Rocco D. Feroci M. (2018). NHC in Imidazolium Acetate Ionic Liquids: Actual or Potential Presence? Front. Chem. 6, 355. 10.3389/fchem.2018.00355 30211149 PMC6121013

[B9] Dai C. Zhang J. Huang C. Lei Z. (2017). Ionic Liquids in Selective Oxidation: Catalysts and Solvents. Chem. Rev. 117, 6929–6983. 10.1021/acs.chemrev.7b00030 28459547

[B10] Ding W. Zhu W. Xiong J. Yang L. Wei A. Zhang M. (2015). Novel Heterogeneous Iron-Based Redox Ionic Liquid Supported on SBA-15 for Deep Oxidative Desulfurization of Fuels. Chem. Eng. J. 266, 213–221. 10.1016/j.cej.2014.12.040

[B11] Doherty S. Knight J. G. Carroll M. A. Clemmet A. R. Ellison J. R. Backhouse T. (2016). Efficient and Selective Oxidation of Sulfides in Batch and Continuous Flow Using Styrene-Based Polymer Immobilised Ionic Liquid Phase Supported Peroxotungstates. RSC Adv. 6, 73118–73131. 10.1039/c6ra11157b

[B12] Doherty S. Knight J. G. Carroll M. A. Ellison J. R. Hobson S. J. Stevens S. (2015). Efficient and Selective Hydrogen Peroxide-Mediated Oxidation of Sulfides in Batch and Segmented and Continuous Flow Using a Peroxometalate-Based Polymer Immobilised Ionic Liquid Phase Catalyst. Green. Chem. 17, 1559–1571. 10.1039/C4GC01770F

[B13] Fareghi-Alamdari R. Zekri N. Moghadam A. J. Farsani M. R. (2017). Green Oxidation of Sulfides to Sulfoxides and Sulfones With H_2_O_2_ Catalyzed by Ionic Liquid Compounds Based on Keplerate Polyoxometalates. Catal. Commun. 98, 71–75. 10.1016/j.catcom.2017.04.050

[B14] Gonçalves D. A. F. Alvim R. P. R. Bicalho H. A. Peres A. M. Binatti I. Batista P. F. R. (2018). Highly Dispersed Mo-Doped Graphite Carbon Nitride: Potential Application as Oxidation Catalyst with Hydrogen Peroxide. New J. Chem. 42, 5720–5727. 10.1039/C8NJ00316E

[B15] Guo Y. Fu J. Li L. Li X. Wang H. Ma W. (2018). One-pot Synthesis of Polyoxomolybdate Anion Intercalated Layered Double Hydroxides and Their Application in Ultra-Deep Desulfurization of Fuels under Mild Conditions. Inorg. Chem. Front. 5, 2205–2210. 10.1039/c8qi00248g

[B16] Gupta R. Yadav M. Gaur R. Arora G. Yadav P. Sharma R. K. (2020). Magnetically Supported Ionic Liquids: a Sustainable Catalytic Route for Organic Transformations. Mater. Horiz. 7, 3097–3130. 10.1039/d0mh01088j

[B17] Hao L. Sun L. Su T. Hao D. Liao W. Deng C. (2019). Polyoxometalate-based Ionic Liquid Catalyst with Unprecedented Activity and Selectivity for Oxidative Desulfurization of Diesel in [Omim]BF_4_ . Chem. Eng. J. 358, 419–426. 10.1016/j.cej.2018.10.006

[B18] Hosseini S. H. Tavakolizadeh M. Zohreh N. Soleyman R. (2018). Green Route for Selective Gram-Scale Oxidation of Sulfides Using Tungstate/triazine-Based Ionic Liquid Immobilized on Magnetic Nanoparticles as a Phase-Transfer Heterogeneous Catalyst. Appl. Organometal Chem. 32, e395. 10.1002/aoc.3953

[B19] Hosseini-Eshbala F. Sedrpoushan A. Breit B. Mohanazadeh F. Veisi H. (2020). Ionic-liquid-modified CMK-3 as a Support for the Immobilization of Molybdate Ions (MoO42-): Heterogeneous Nanocatalyst for Selective Oxidation of Sulfides and Benzylic Alcohols. Mater. Sci. Eng. C. 110, 110577. 10.1016/j.msec.2019.110577 32204056

[B20] Hu Y.-L. Fang D. Xing R. (2014a). Efficient and Convenient Oxidation of Sulfides to Sulfoxides with Molecular Oxygen Catalyzed by Mn(OAc)_2_ in Ionic Liquid [C_12_mim][NO_3_]. RSC Adv. 4, 51140–51145. 10.1039/c4ra06695b

[B21] Hu Y.-L. Liu X.-B. Fang D. (2014b). Efficient and Convenient Oxidation of Sulfides to Sulfones Using H_2_O_2_catalyzed by V2O5in Ionic Liquid [C_12_mim][HSO_4_]. Catal. Sci. Technol. 4, 38–42. 10.1039/c3cy00719g

[B22] Huang W. Zhu W. Li H. Shi H. Zhu G. Liu H. (2010). Heteropolyanion-based Ionic Liquid for Deep Desulfurization of Fuels in Ionic Liquids. Ind. Eng. Chem. Res. 49, 8998–9003. 10.1021/ie100234d

[B23] Jiang W. Dong L. Li H. Jia H. Zhu L. Zhu W. (2019a). Magnetic Supported Ionic Liquid Catalysts with Tunable Pore Volume for Enhanced Deep Oxidative Desulfurization. J. Mol. Liquids. 274, 293–299. 10.1016/j.molliq.2018.10.069

[B24] Jiang W. Xiao J. Dong L. Wang C. Li H. Luo Y. (2019b). Polyoxometalate-based Poly(ionic Liquid) as a Precursor for Superhydrophobic Magnetic Carbon Composite Catalysts toward Aerobic Oxidative Desulfurization. ACS Sustainable Chem. Eng. 7, 15755–15761. 10.1021/acssuschemeng.9b04026

[B25] Julião D. Gomes A. C. Pillinger M. Valença R. Ribeiro J. C. Gonçalves I. S. (2016). A Recyclable Ionic Liquid-Oxomolybdenum(VI) Catalytic System for the Oxidative Desulfurization of Model and Real Diesel Fuel. Dalton Trans. 45, 15242–15248. 10.1039/c6dt02065h 27603728

[B26] Karimi B. Khorasani M. Bakhshandeh Rostami F. Elhamifar D. Vali H. (2015). Tungstate Supported on Periodic Mesoporous Organosilica with Imidazolium Framework as an Efficient and Recyclable Catalyst for the Selective Oxidation of Sulfides. ChemPlusChem. 80, 990–999. 10.1002/cplu.201500010 31973256

[B27] Kore R. Berton P. Kelley S. P. Aduri P. Katti S. S. Rogers R. D. (2017). Group IIIA Halometallate Ionic Liquids: Speciation and Applications in Catalysis. ACS Catal. 7, 7014–7028. 10.1021/acscatal.7b01793

[B28] Lethesh K. C. Evjen S. Raj J. J. Roux D. C. D. Venkatraman V. Jayasayee K. (2019). Hydroxyl Functionalized Pyridinium Ionic Liquids: Experimental and Theoretical Study on Physicochemical and Electrochemical Properties. Front. Chem. 7, 625. 10.3389/fchem.2019.00625 31620423 PMC6759651

[B29] Li T. Zhang W. Chen W. Miras H. N. Song Y.-F. (2018). Modular Polyoxometalate-Layered Double Hydroxides as Efficient Heterogeneous Sulfoxidation and Epoxidation Catalysts. ChemCatChem. 10 (1), 188–197. 10.1002/cctc.201701056

[B30] Li Y. Zhang Y. Wu P. Feng C. Xue G. (2018). Catalytic Oxidative/Extractive Desulfurization of Model Oil Using Transition Metal Substituted Phosphomolybdates-Based Ionic Liquids. Catalysts. 8, 639. 10.3390/catal8120639

[B31] Marinkovic J. M. Riisager A. Franke R. Wasserscheid P. Haumann M. (2019). Fifteen Years of Supported Ionic Liquid Phase-Catalyzed Hydroformylation: Material and Process Developments. Ind. Eng. Chem. Res. 58, 2409–2420. 10.1021/acs.iecr.8b04010

[B32] Moaser A. G. Ahadi A. Rouhani S. Mamba B. B. Msagati T. A.-M. Rostamnia S. (2020). Curbed of Molybdenum Oxido-Diperoxido Complex on Ionic Liquid Body of Mesoporous Bipy-PMO-IL as a Promising Catalyst for Selective Sulfide Oxidation. J. Mol. Liquids. 312, 113388. 10.1016/j.molliq.2020.113388

[B33] Mohumed H. Rahman S. Imtiaz S. A. Zhang Y. (2020). Oxidative-extractive Desulfurization of Model Fuels Using a Pyridinium Ionic Liquid. ACS Omega. 5, 8023–8031. 10.1021/acsomega.0c00096 32309712 PMC7161041

[B34] Mojaverian Kermani A. Mahmoodi V. Ghahramaninezhad M. Ahmadpour A. (2021). Highly Efficient and green Catalyst of {Mo132} Nanoballs Supported on Ionic Liquid-Functionalized Magnetic Silica Nanoparticles for Oxidative Desulfurization of Dibenzothiophene. Separation Purif. Technology. 258, 117960. 10.1016/j.seppur.2020.117960

[B35] Nasrollahzadeh M. Ghasemzadeh M. Gharoubi H. Nezafat Z. (2021). Progresses in Polysaccharide and Lignin-Based Ionic Liquids: Catalytic Applications and Environmental Remediation. J. Mol. Liquids. 342, 117559. 10.1016/j.molliq.2021.117559

[B36] Pourjavadi A. Nazari-Chamazkoti M. Hosseini S. H. (2015). Polymeric Ionic Liquid Nanogel-Anchored Tungstate Anions: a Robust Catalytic System for Oxidation of Sulfides to Sulfoxides. New J. Chem. 39, 1348–1354. 10.1039/c4nj01931h

[B37] Rafiee E. Mirnezami F. (2014). Keggin-Structured Polyoxometalate-Based Ionic Liquid Salts: Thermoregulated Catalysts for Rapid Oxidation of Sulfur-Based Compounds Using H_2_O_2_ and Extractive Oxidation Desulfurization of Sulfur-Containing Model Oil. J. Mol. Liquids. 199, 156–161. 10.1016/j.molliq.2014.08.036

[B38] Rafiee E. Shahebrahimi S. (2017). Organic-inorganic Hybrid Polyionic Liquid Based Polyoxometalate as Nano Porous Material for Selective Oxidation of Sulfides. J. Mol. Struct. 1139, 255–263. 10.1016/j.molstruc.2017.03.041

[B39] Raiguel S. Dehaen W. Binnemans K. (2020). Stability of Ionic Liquids in Brønsted-Basic media. Green. Chem. 22, 5225–5252. 10.1039/d0gc01832e

[B40] Rajabi F. Vessally E. Luque R. Voskressensky L. (2021). Highly Efficient and Selective Aqueous Aerobic Oxidation of Sulfides to Sulfoxides or Sulfones Catalyzed by Tungstate-Functionalized Nanomaterial. Mol. Catal. 515, 111931. 10.1016/j.mcat.2021.111931

[B41] Rajeswari M. Lumb A. Khurana J. M. (2016). The Highly Selective Metal-Free Oxidation of Sulfides, Tellurides and Phosphines Using Sodium Bromate in the Presence of Recyclable Ionic Liquid [Bmim]HSO4, at 80 °C. J. Chem. Res. 40, 442–444. 10.3184/174751916x14656595396325

[B42] Ren Z. Zhou Z. Li M. Zhang F. Wei L. Liu W. (2019). Deep Desulfurization of Fuels Using Imidazole Anion-Based Ionic Liquids. ACS Sustainable Chem. Eng. 7, 1890–1900. 10.1021/acssuschemeng.8b03566

[B43] Rostamnia S. Gholipour B. Golchin Hosseini H. (2016). Metal- and Halogen-free Hydrogensulfate Ionic liquid/SBA-15 as Catalyst in Clean Oxidation of Aromatic and Aliphatic Organic Sulfides with Aqueous Hydrogen Peroxide. Process Saf. Environ. Prot. 100, 74–79. 10.1016/j.psep.2015.12.009

[B44] Saikia B. K. Khound K. Baruah B. P. (2014). Extractive De-sulfurization and De-Ashing of High Sulfur Coals by Oxidation with Ionic Liquids. Energ. Convers. Management 81, 298–305. 10.1016/j.enconman.2014.02.043

[B45] Sedrpoushan A. Hosseini‐Eshbala F. Mohanazadeh F. Heydari M. (2018). Tungstate Supported Mesoporous Silica SBA‐15 with Imidazolium Framework as a Hybrid Nanocatalyst for Selective Oxidation of Sulfides in the Presence of Hydrogen Peroxide. Appl. Organometal Chem. 32, e4004. 10.1002/aoc.4004

[B46] Shao B.-b. Shi L. Meng X. (2014). Deep Desulfurization of 4,6-dimethyldienzothiophene by an Ionic Liquids Extraction Coupled with Catalytic Oxidation with a Molybdic Compound. Ind. Eng. Chem. Res. 53, 6655–6663. 10.1021/ie500236b

[B47] Tarkhanova I. G. Anisimov A. V. Buryak A. K. Bryzhin A. A. Ali-Zade A. G. Akopyan A. V. (2017). Immobilized Ionic Liquids Based on Molybdenum- and Tungsten-Containing Heteropoly Acids: Structure and Catalytic Properties in Thiophene Oxidation. Pet. Chem. 57 (10), 859–867. 10.1134/s0965544117100164

[B48] Tarkhanova I. G. Anisimov A. V. Verzhichinskaya S. V. Buryak A. K. Zelikman V. M. Gantman M. G. (2016). Immobilized Copper- and Molybdenum-Containing Ionic Liquids in Oxidation of Sulfides. Pet. Chem. 56, 158–165. 10.1134/s0965544116020158

[B49] Ternois J. Guillen F. Plaquevent J.-C. Coquerel G. (2007). Asymmetric Synthesis of Modafinil and its Derivatives by Enantioselective Oxidation of Thioethers: Comparison of Various Methods Including Synthesis in Ionic Liquids. Tetrahedron: Asymmetry. 18, 2959–2964. 10.1016/j.tetasy.2007.11.031

[B50] Venkat Reddy C. Verkade J. G. (2007). An Advantageous Tetrameric Titanium Alkoxide/Ionic Liquid as a Recyclable Catalyst System for the Selective Oxidation of Sulfides to Sulfones. J. Mol. Catal. A: Chem. 272, 233–240. 10.1016/j.molcata.2007.02.053

[B51] Wang C. Chen Z. Yao X. Jiang W. Zhang M. Li H. (2017a). One-pot Extraction and Aerobic Oxidative Desulfurization with Highly Dispersed V_2_O_5_/SBA-15 Catalyst in Ionic Liquids. RSC Adv. 7, 39383–39390. 10.1039/c7ra07286d

[B52] Wang C. Chen Z. Zhu W. Wu P. Jiang W. Zhang M. (2017b). One-pot Extraction and Oxidative Desulfurization of Fuels with Molecular Oxygen in Low-Cost Metal-Based Ionic Liquids. Energy Fuels. 31, 1376–1382. 10.1021/acs.energyfuels.6b02624

[B53] Wang J. Zhang L. Sun Y. Jiang B. Chen Y. Gao X. (2018). Deep Catalytic Oxidative Desulfurization of Fuels by Novel Lewis Acidic Ionic Liquids. Fuel Process. Technology. 177, 81–88. 10.1016/j.fuproc.2018.04.013

[B54] Wang L. Jin G. Xu Y. (2019). Desulfurization of Coal Using Four Ionic Liquids with [HSO_4_]−. Fuel. 236, 1181–1190. 10.1016/j.fuel.2018.09.082

[B55] Wang S. Wang L. Đaković M. Popović Z. Wu H. Liu Y. (2012). Bifunctional Ionic Liquid Catalyst Containing Sulfoacid Group and Hexafluorotitanate for Room Temperature Sulfoxidation of Sulfides to Sulfoxides Using Hydrogen Peroxide. ACS Catal. 2, 230–237. 10.1021/cs200501n

[B56] Xin B. Hao J. (2014). Imidazolium-Based Ionic Liquids Grafted on Solid Surfaces. Chem. Soc. Rev. 43, 7171–7187. 10.1039/c4cs00172a 25000475

[B57] Xu H. Zhang J. Zhang D. Guo Y. Wu F. (2021). Catalytic Oxidation Desulfurization of Silica-Gel-Supported Ionic Liquid [Bmim]CoCl_3_ Coupling Oxone. Fuel. 288, 119655. 10.1016/j.fuel.2020.119655

[B58] Xun S. Ti Q. Wu L. He M. Chen L. Yang W. (2020). Few Layer G-C3n4 Dispersed Quaternary Phosphonium Ionic Liquid for Highly Efficient Catalytic Oxidative Desulfurization of Fuel. Energy Fuels. 34, 12379–12387. 10.1021/acs.energyfuels.0c02357

[B59] Xun S. Zhu W. Zheng D. Li H. Jiang W. Zhang M. (2015). Supported Ionic Liquid [Bmim]FeCl_4_/Am TiO_2_ as an Efficient Catalyst for the Catalytic Oxidative Desulfurization of Fuels. RSC Adv. 5, 43528–43536. 10.1039/c5ra00999e

[B60] Yang H. Jiang B. Sun Y. Zhang L. Sun Z. Wang J. (2017). Polymeric Cation and Isopolyanion Ionic Self-Assembly: Novel Thin-Layer Mesoporous Catalyst for Oxidative Desulfurization. Chem. Eng. J. 317, 32–41. 10.1016/j.cej.2017.01.135

[B61] Yuan J. Xiong J. Wang J. Ding W. Yang L. Zhang M. (2016). Structure and Catalytic Oxidative Desulfurization Properties of SBA-15 Supported Silicotungstic Acid Ionic Liquid. J. Porous Mater. 23, 823–831. 10.1007/s10934-016-0137-8

[B62] Zhang B. Li S. Yue S. Cokoja M. Zhou M.-D. Zang S.-L. (2013). Imidazolium Perrhenate Ionic Liquids as Efficient Catalysts for the Selective Oxidation of Sulfides to Sulfones. J. Organomet. Chem. 744, 108–112. 10.1016/j.jorganchem.2013.05.043

[B63] Zhang B. Zhou M.-D. Cokoja M. Mink J. Zang S.-L. Kühn F. E. (2012). Oxidation of Sulfides to Sulfoxides Mediated by Ionic Liquids. RSC Adv. 2, 8416–8420. 10.1039/C2RA21323K

[B64] Zhang H. Qi L. (2018). A Novel Catalytic System Poly(1-Vinyl-3-Dodecylimidazolium Tribromide)/TBN for the Oxidation of Sulfides to Sulfoxides with Air as Oxidant. Tetrahedron Lett. 59, 3171–3174. 10.1016/j.tetlet.2018.07.001

[B65] Zhang L. Wang J. Sun Y. Jiang B. Yang H. (2017). Deep Oxidative Desulfurization of Fuels by Superbase-Derived Lewis Acidic Ionic Liquids. Chem. Eng. J. 328, 445–453. 10.1016/j.cej.2017.07.060

[B66] Zhang Y. Tan R. Gao M. Hao P. Yin D. (2017). Bio-inspired Single-Chain Polymeric Nanoparticles Containing a Chiral Salen TiIV Complex for Highly Enantioselective Sulfoxidation in Water. Green. Chem. 19, 1182–1193. 10.1039/c6gc02743a

[B67] Zhao H. Baker G. A. (2015). Oxidative Desulfurization of Fuels Using Ionic Liquids: A Review. Front. Chem. Sci. Eng. 9, 262–279. 10.1007/s11705-015-1528-0 33907629 PMC8075298

[B68] Zhao P. Zhang M. Wu Y. Wang J. (2012). Heterogeneous Selective Oxidation of Sulfides with H_2_O_2_ Catalyzed by Ionic Liquid-Based Polyoxometalate Salts. Ind. Eng. Chem. Res. 51, 6641–6647. 10.1021/ie202232j

[B69] Zhao W. Yang C. Huang J. Jin X. Deng Y. Wang L. (2020). Selective Aerobic Oxidation of Sulfides to Sulfoxides in Water under Blue Light Irradiation over Bi_4_O_5_Br_2_ . Green. Chem. 22, 4884–4889. 10.1039/d0gc01930e

[B70] Zhou Q. Ye M. Ma W. Li D. Ding B. Chen M. (2019). Ionic Liquid Stabilized Niobium Oxoclusters Catalyzing Oxidation of Sulfides with Exceptional Activity. Chem. Eur. J. 25, 4206–4217. 10.1002/chem.201806178 30690807

