# Supplementary material for: Recent Advances in Catalytic Oxidation of Organic Sulfides: Applications of Metal–Ionic Liquid Catalytic Systems
Source: Front Chem. 2022 Feb 28;9:798603. doi: 10.3389/fchem.2021.798603 (PMC8918828; doi:10.3389/fchem.2021.798603)
Supplement: Supplementary file 1 [file DataSheet1.doc]

**Supporting Information**

**Recent Advances in Catalytic Oxidation of Organic Sulfides Applications of Metal-Ionic Liquid Catalytic Systems**

Xiao Bing Liu^1^, Qi Rong^2^, Jin Tan^2^, Chen Chen^3^, Yu Lin Hu^2*^

^1^College of Chemistry and Chemical Engineering, Jinggangshan University, Ji'an 343009, P. R. China

^2^Key laboratory of inorganic nonmetallic crystalline and energy conversion materials, College of Materials and Chemical Engineering, China Three Gorges University, Yichang 443002, P. R. China

^3^College of Environmental and Chemical Engineering, Jiangsu University of Science and Technology, Zhenjiang 212003, P. R. China

*Correspondence to: E-mail: [huyulin1982@163.com](mailto:huyulin1982@163.com)

**SCHEME S1.** Preparation of POM-ILs and catalytic oxidation of sulfides to sulfoxides (Rafiee et al. 2014).

**SCHEME S2**. Structures of ionic liquid anions and catalytic oxidation of methyl phenyl sulfide (Bigi, et al. 2011).

**SCHEME S3.** Catalytic oxidation of sulfides to corresponding sulfoxides by H_2_O_2_ in the presence of Mo132-MimAM (Fareghi-Alamdari, et al. 2017).

**SCHEME S4.** Synthesis of the PVPyPSPMo_10_V_2_ catalyst and catalytic sulfides oxidation (Rafiee et al. 2017).

**SCHEME S5.** Selective oxidation of sulfides to sulfoxides with hydrogen peroxide catalyzed by [PO_4_{WO(O_2_)_2_}_4_]@ImPIILP (3a-e) (Doherty, et al. 2016).

**SCHEME S6.** (a) Imidazolium-based styrene monomers (b) polystyrene-based ionic co-polymers (X =Cl^-^, Br^-^) used for the preparation of POM@ImPIILP 3a-c (X= [PO_4_{WO(O_2_)_2_ }_4_]^3-^) (c) macroreticular resin 2d and POM@PIILP 3d and (d) imidazolium-modified Merrifield resin and POM@ImPIILP 3e (Doherty, et al. 2016).

**SCHEME S7.**  Preparation of PILW catalyst and catalytic oxidation of sulfides to sulfoxides (Pourjavadi et al. 2015).

**SCHEME S8**. Synthesis of [PO_4_{WO(O_2_)_2_}_4_]@PIILP and selective oxidation of sulphides with hydrogen peroxide (Doherty et al. 2015).

**SCHEME S9**. Synthesis of immobilized metal-containing ionic liquids and catalytic oxidation of diethyl sulfide and methyl phenyl sulfide (Tarkhanova, et al. 2016).

**SCHEME S10.** Synthesis of mineral supported ionic liquids (*m* = 2-5; *n* = 7–13; *l* = 2-7; and *k* = 4-20) (Tarkhanova et al. 2017).

**SCHEME S11**. Catalytic oxidation of sulfides to sulfoxides with Mg_3_Al-ILs-La(PW_11_)_2_ (Li et al. 2018).

**SCHEME S12.** Oxidation of sulfides to sulfones catalyzed by WO_4_^2−^@PMO-IL (Karimi et al. 2015).

**SCHEME S13**. Preparation of SBA-15+ImCl+MoO_5_ and its catalytic oxidation of sulfides (Carrasco et al. 2015).


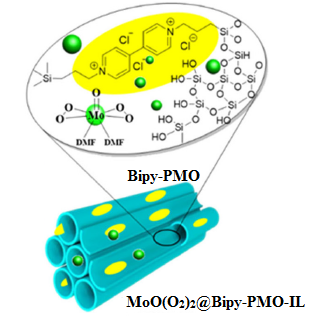


**SCHEME S14.** Catalytic oxidation of sulfides to sulfoxides with MoO(O_2_)_2_@Bipy-PMO-IL (Moaser et al. 2020).

#

# SCHEME S15. Catalytic oxidation of sulfides to sulfoxides with H_2_O_2_ using CMK-3-OctIm/MoO_4_= (**[Hosseini-Eshbala](https://www.sciencedirect.com/science/article/pii/S0928493119332060#!)** et al. 2020).


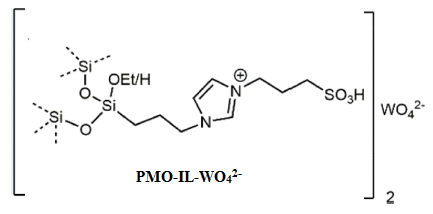


# SCHEME S16. Catalytic aerobic oxidation of sulfides to sulfoxides or sulfones with PMO-IL-WO_4_^2-^(**[Rajabi](https://www.sciencedirect.com/science/article/pii/S2468823121005484#!)** et al. 2021).

**SCHEME S17**. Catalytic reaction pathway of ECODS for DBT (Hao et al. 2019).

**SCHEME S18**. Oxidation of organic sulfur compounds in IL medium.

**SCHEME S19.** Synthetic route for the catalysts [HDBN]Cl/nZnCl_2_.

**SCHEME S20.** Synthetic route for [ODBU]Cl/nZnCl_2_.

**SCHEME S21.** Catalytic process of the oxidative desulfurization system.

**SCHEME S22.** The synthetic process of [pmim]FeCl_4_-SBA-15.


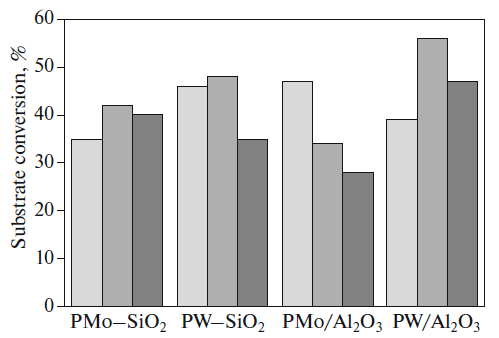


**FIGURE S1.** Catalytic performance of the catalysts immobilized on Perlkat and γ-Al_2_O_3_ in three consecutive cycles (Tarkhanova et al. 2017).


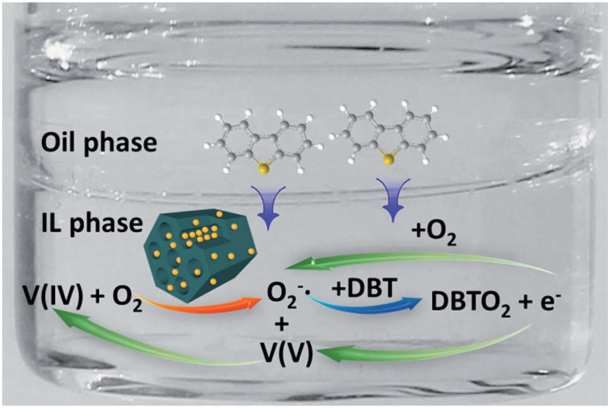


**FIGURE S2.** Catalytic process for the oxidative desulfurization (Wang et al. 2017).

**FIGURE S3.** Recycling of [HDBN]Cl/ZnCl_2_ (Zhang et al. 2017).

**FIGURE S4**. ^1^H NMR analysis of the (a) fresh IL and (b) recycled ILs (Wang et al. 2018).

**FIGURE S5**. Recycling of [Hnmp]Cl/ZnCl_2_ on S-removal in the ODS process (Chen et al. 2015).

**
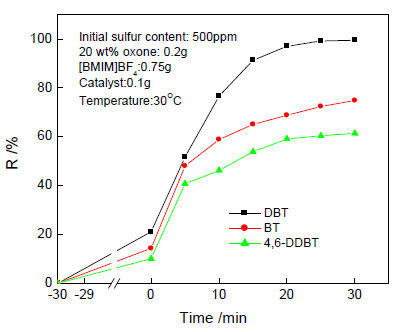

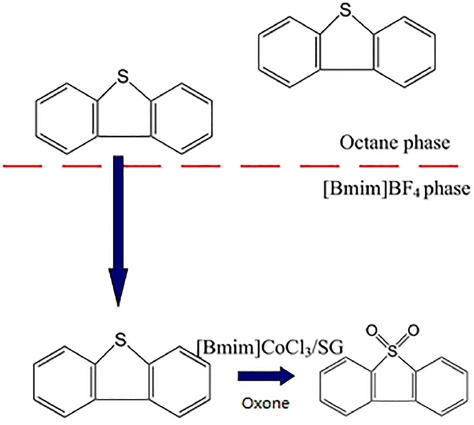
**

**FIGURE S6**. Effect of sulfur species on the ECODS process and the catalytic oxidation desulfurization process (Xu et al. 2021).

**FIGURE S7.** Catalytic oxidative desulfurization with [Bmim]FeCl_4_/Am TiO_2_ (Xun et al. 2015).


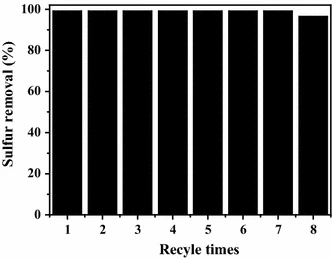


**FIGURE S8.** Recycle of the catalytic system for the oxidative desulfurization of fuels (Yuan et al. 2016).

**FIGURE S9**. Sulfur removal of different sulfur species (Ding et al. 2015).

**
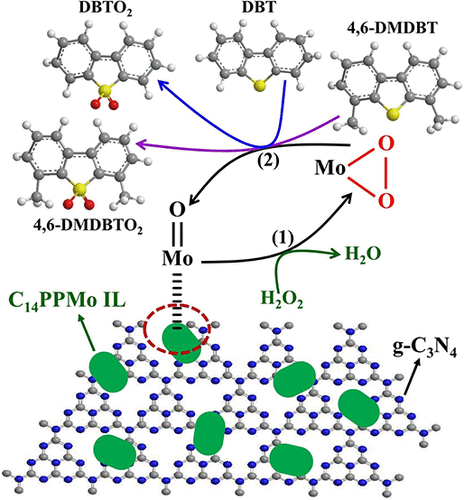
**

**FIGURE S10**. Catalytic process for the oxidation desulfurization system (Xun et al. 2020).
